# Relationship between preoperative hemoglobin A1c and late postoperative coronary flow reserve improvement after coronary artery bypass grafting

**DOI:** 10.1007/s11748-025-02189-0

**Published:** 2025-08-12

**Authors:** Takahiro Fujimoto, Kentaro Honda, Hideki Kunimoto, Ryo Nakamura, Mizuho Ikuchi, Yuya Ideguchi, Kota Agematsu, Yoshiharu Nishimura

**Affiliations:** Department of Thoracic and Cardiovascular Surgery, Wakayama Medical University, 811-1, Kimiidera, Wakayama, 641-8509 Japan

**Keywords:** Hemoglobin A1c, Coronary artery bypass grafting, Coronary flow reserve

## Abstract

**Objective:**

This study was performed to investigate the relationship between the preoperative hemoglobin A1c concentration and late postoperative coronary flow reserve improvement after coronary artery bypass grafting.

**Methods:**

The data of 61 patients who underwent isolated coronary artery bypass grafting were retrospectively analyzed. Coronary flow reserve was measured preoperatively, in the early postoperative period (mean: 2.5 months), and in the late postoperative period (mean: 25 months). The patients were classified into two groups based on their preoperative hemoglobin A1c concentration: Group N (< 7%) and Group D (≥ 7%). Further classification was based on the duration of diabetes mellitus (< 10 years or ≥ 10 years).

**Results:**

There was no significant difference in early postoperative coronary flow reserve between the two groups. However, in the late postoperative period, Group N exhibited significantly greater coronary flow reserve improvement than Group D (*p* = 0.012). There was a significant correlation between a lower preoperative hemoglobin A1c concentration and greater late postoperative coronary flow reserve improvement (*R*^*2*^ = 0.13, *p* = 0.019). Patients with a longer history of diabetes mellitus and a higher preoperative hemoglobin A1c concentration had poorer coronary flow reserve improvement in the late postoperative period.

**Conclusions:**

The preoperative hemoglobin A1c concentration predicted coronary flow reserve improvement in the late postoperative period after coronary artery bypass grafting.

**Supplementary Information:**

The online version contains supplementary material available at 10.1007/s11748-025-02189-0.

## Introduction

Patients with diabetes mellitus (DM) are known to have coronary microcirculation impairment [1], and around half of patients who undergo coronary artery bypass grafting (CABG) have a history of DM [2]. Coronary flow reserve (CFR) is used as an indicator of the microcirculation, and a previous study showed that CFR is decreased in patients with DM [3]. CABG improves CFR to a greater degree than percutaneous coronary intervention [4]. CFR is also reportedly related to major adverse cardiovascular events; therefore, it is important to improve CFR after surgery [5]. Whether CABG improves CFR in the late postoperative period and the possible factors that predict such an improvement remain unclear.

Guidelines for DM propose a target hemoglobin A1c (HbA1c) concentration for the prevention of microvascular complications [6]. Patients with DM with poor HbA1c control may develop ischemic heart disease, and CABG is one of the most effective treatments for these patients. However, how preoperative HbA1c control affects the coronary microcirculation in the late postoperative period after CABG has not been fully investigated. We hypothesized that a higher preoperative HbA1c concentration would predict impaired late postoperative CFR improvement after CABG.

### Objective

The present study was performed to determine how the preoperative HbA1c concentration affects the coronary microcirculation (measured by CFR) after CABG.

## Subjects

This retrospective study involved 61 patients who underwent isolated CABG at Wakayama Medical University Hospital from October 2010 to December 2020. All patients had confirmed graft patency and underwent CFR measurements preoperatively, in the early postoperative period, and in the late postoperative period. Patients who underwent hemodialysis were excluded. Of the 61 patients, 77% were male. The mean age of the patients was 66 ± 9.0 years. CFR was calculated by measuring the flow velocity of the left anterior descending branch by transthoracic echocardiography. CFR was defined as the ratio of the coronary artery blood flow velocity during maximal coronary artery dilation divided by the resting coronary artery blood flow velocity. The blood flow velocity during maximal coronary artery dilation was measured at the time of maximal blood flow after continuous administration of adenosine triphosphate at 0.14 mg/kg/min. Early postoperative CFR and late postoperative CFR were measured at 2.5 ± 3.1 months and 25 ± 22 months postoperatively, respectively.

## Methods

The patients were classified into two groups according to their preoperative HbA1c concentration. In accordance with the 2019 Diabetes Care Guidelines, the HbA1c threshold for the prevention of complications was < 7% [7]. Group N comprised patients with an HbA1c concentration of < 7% (*n* = 39), and Group D comprised patients with an HbA1c concentration of ≥ 7% (*n* = 22). In addition, the patients were further classified according to the duration of DM: ≥ 10 years (Group N: *n* = 9, Group D: *n* = 17) or < 10 years (Group N: *n* = 29, Group D: *n* = 5). Only one patient in Group N had an unknown history of DM and was excluded. The degree of improvement in late postoperative CFR was compared between the groups. The late postoperative CFR improvement ratio was calculated as (late postoperative CFR − early postoperative CFR) ÷ early postoperative CFR. CFR depends on both epicardial vessels and the microcirculation [8]. After CABG, however, epicardial vascular lesions are almost negligible, making CFR a suitable method for evaluating the postoperative coronary microcirculation.

Multiple regression analysis was then performed using the continuous variable, namely the late postoperative CFR improvement rate, as the objective variable. The explanatory variables were low-density lipoprotein cholesterol, left ventricular mass index, ejection fraction, and preoperative HbA1c, all of which may influence CFR. These explanatory variables have previously been reported to be correlated with CFR [9–11].

Additionally, when the objective variable was non-improvement in late postoperative CFR (defined as a postoperative CFR improvement rate of ≤ 0), the explanatory variables were male sex, hypertension, dyslipidemia (low-density lipoprotein cholesterol of ≥ 120 mg/dL), chronic kidney disease (estimated glomerular filtration rate of < 60 mL/min/1.73 m^2^), diabetes mellitus (preoperative HbA1c of ≥ 7%), old myocardial infarction, ejection fraction of < 50%, left ventricular hypertrophy (left ventricular mass index of ≥ 115 g/m^2^ for men, ≥ 95 g/m^2^ for women), postoperative use of beta blockers, treatment with angiotensin-converting enzyme inhibitors or angiotensin receptor blockers, treatment with metformin, treatment with dipeptidyl peptidase-4 inhibitors, treatment with insulin, and late postoperative HbA1c improvement. Late postoperative HbA1c improvement was defined as a decrease in HbA1c from the preoperative level to the time of CFR measurement in the late postoperative period (i.e., [HbA1c at the time of CFR measurement] − [preoperative HbA1c] < 0%). Univariate analysis (logistic regression analysis) was also performed.

The threshold values were determined as follows. The LDL-C cutoff of 120 mg/dL was based on the lipid management targets for high-risk patients outlined in the Japan Atherosclerosis Society Guidelines 2022 [12]. The eGFR threshold was set according to the KDIGO 2024 CKD Guidelines [13]. The EF cutoff was selected with reference to the threshold used by Hyun et al. [9], who investigated the relationship between EF and CFR. Left ventricular hypertrophy was defined in accordance with the current echocardiographic chamber quantification guidelines [14].

### Statistical analysis

Continuous variables are expressed as mean ± standard deviation and were compared between two groups using the t-test. Categorical variables are presented as frequencies and were analyzed using the Chi-square test. One-way analysis of variance was used to compare four groups. Significant differences are expressed as *p* < 0.05. Excel (Microsoft Corp., Redmond, WA, USA) was used for the statistical analyses.

## Results

### Preoperative data

The patients’ background characteristics before CABG in both groups are shown in Table 1. The patients in Group D were taking significantly more DM medications, including insulin. Furthermore, the ejection fraction was lower in Group D than in Group N (*p* < 0.05). Moreover, old myocardial infarction was more frequent in Group D than in Group N (*p* < 0.01).

**Table 1:** Baseline and clinical characteristics of the included patients

Variables	Group N (*n* = 39)	Group D (*n* = 22)	*p* value
Age, years	65.8 ± 9.6	66.7 ± 8.2	0.44
Male sex	29 (74)	18 (82)	0.071
BSA, m^2^	1.7 ± 0.19	1.7 ± 0.14	0.94
BMI, kg/m^2^	23.4 ± 3.5	23.4 ± 3.0	0.99
Smoking	25 (64)	15 (68)	0.37
Hypertension	36 (92)	21 (96)	0.32
DLP	8.0 (21)	3.0 (14)	0.25
CKD	13 (33)	8.0 (36)	0.41
HbA1c, %	5.9 ± 0.46	7.7 ± 0.68	< 0.001
Medications			
Aspirin	33 (85)	17 (77)	0.24
β-Blocker	21 (54)	16 (73)	0.074
Calcium blocker	13 (33)	6 (27)	0.31
ACEI/ARB	26 (67)	13 (59)	0.28
Statin	27 (69)	22 (82)	0.14
MRA	3 (7.7)	0 (0)	0.10
Metformin	1 (2.6)	4 (18)	0.016
Sulfonylurea	3 (7.7)	6 (27)	0.019
DPP-4 inhibitor	2 (5.1)	9 (41)	< 0.001
SGLT2 inhibitor	0 (0)	2 (9)	0.028
α-Glucosidase inhibitor	1 (2.6)	3 (14)	0.047
Insulin	5 (13)	15 (68)	< 0.001
Echocardiographic findings			
Ejection fraction, %	54.3 ± 6.8	49.3 ± 9.2	0.018
LV mass index	86.7 ± 19.8	82.3 ± 21.2	0.69
E-velocity, cm/s	60.7 ± 16.0	60.2 ± 15.0	0.91
A-velocity, m/s	74.6 ± 17.8	76.0 ± 22.4	0.78
E/A	0.85 ± 0.32	0.87 ± 0.34	0.87
Deceleration time, ms	258 ± 55.8	250 ± 73.7	0.62
E/E'	10.8 ± 4.2	10.7 ± 3.5	0.91
OMI	19 (49)	16 (80)	0.0083
Coronary angiography findings			
LAD CTO	5.0 (13)	1.0 (4.6)	0.15

Data are presented as mean ± standard deviation or n (%)

*BSA* body surface area, *BMI* body mass index, *DLP* dyslipidemia (low-density lipoprotein cholesterol concentration ≥ 120 mg/dL), *CKD* chronic kidney disease, *ACEI/ARB* angiotensin-converting enzyme inhibitor/angiotensin II receptor blocker, *MRA* mineralocorticoid receptor antagonist, *DPP-4 inhibitor* dipeptidyl peptidase-4 inhibitor, *SGLT2* sodium–glucose co-transporter-2, *LV* left ventricular, *OMI* old myocardial infarction, *LAD CTO* left anterior descending artery chronic total occlusion

### Intraoperative data

Table 2 presents the intraoperative data. There were no significant differences in the surgical procedures. There was also no significant difference in graft blood flow, indicating no difference in bypass quality.

**Table 2:** Intraoperative data

Intraoperative data	Group N (*n* = 39)	Group D (*n* = 22)	*p* value
On pump	3 (7.7)	3 (14)	0.23
Distal anastomoses, n	3.3 ± 1.2	3.9 ± 1.0	0.063
Transit time flow meter^a^			
Mean graft flow	30.8 ± 16.4	27.0 ± 17.8	0.41
Pulsatility index	2.2 ± 0.82	2.7 ± 1.2	0.051

Data are presented as mean ± standard deviation or n (%)

*LITA* left internal thoracic artery, *IABP* intra-aortic balloon pump

^a^Device that assesses graft quality by measuring intraoperative graft flow

### Late postoperative data

Table 3 provides information on the patients’ medications and echocardiographic findings in the late postoperative period. Regarding medications, DM medications were more common in Group D than in Group N, similar to the preoperative period. Angiotensin-converting enzyme inhibitor/angiotensin II receptor blocker therapy was significantly less common in Group D (*p* < 0.01), with no significant differences in the use of other medications. No significant difference was seen in the echocardiography findings.

**Table 3:** Medications and echocardiographic findings in the late postoperative period

Late postoperative data	Group N (*n* = 39)	Group D (*n* = 22)	*p* value
Medications			
Aspirin	35 (89.7)	22 (100)	0.060
β-Blocker	25 (64)	14 (64)	0.49
Calcium blocker	19 (49)	13 (59)	0.22
ACEI/ARB	17 (44)	3 (14)	0.0084
Statin	25 (64)	13 (59)	0.35
MRA	5 (13)	3 (14)	0.46
Metformin	1 (2.6)	7 (32)	< 0.001
Sulfonylurea	3 (7.7)	6 (27)	0.019
DPP-4 inhibitor	5 (13)	12 (55)	< 0.001
SGLT2 inhibitor	0 (0)	4 (18)	0.0029
α-Glucosidase inhibitor	0 (0)	2 (9)	0.028
Insulin	4 (10)	11 (50)	< 0.001
Echocardiographic findings			
Ejection fraction, %	54.6 ± 7.0	52.1 ± 6.5	0.19
LV mass index	81.6 ± 16.8	82.9 ± 26.1	0.82
E-velocity, cm/s	60.3 ± 15.9	67.8 ± 18.0	0.10
A-velocity, m/s	72.5 ± 16.4	81.9 ± 20.7	0.064
E/A	0.86 ± 0.27	0.87 ± 0.26	0.87
Deceleration time, ms	279.6 ± 71.8	245.1 ± 110.8	0.16
E/E'	8.9 ± 2.3	10.4 ± 4.0	0.072

Data are presented as mean ± standard deviation or n (%)

*ACEI/ARB* angiotensin-converting enzyme inhibitor/angiotensin II receptor blocker, *MRA* mineralocorticoid receptor antagonist, *DPP-4 inhibitor* dipeptidyl peptidase-4 inhibitor, *SGLT2* sodium–glucose co-transporter-2, *LV* left ventricular

### CFR data

**Fig. 1:**
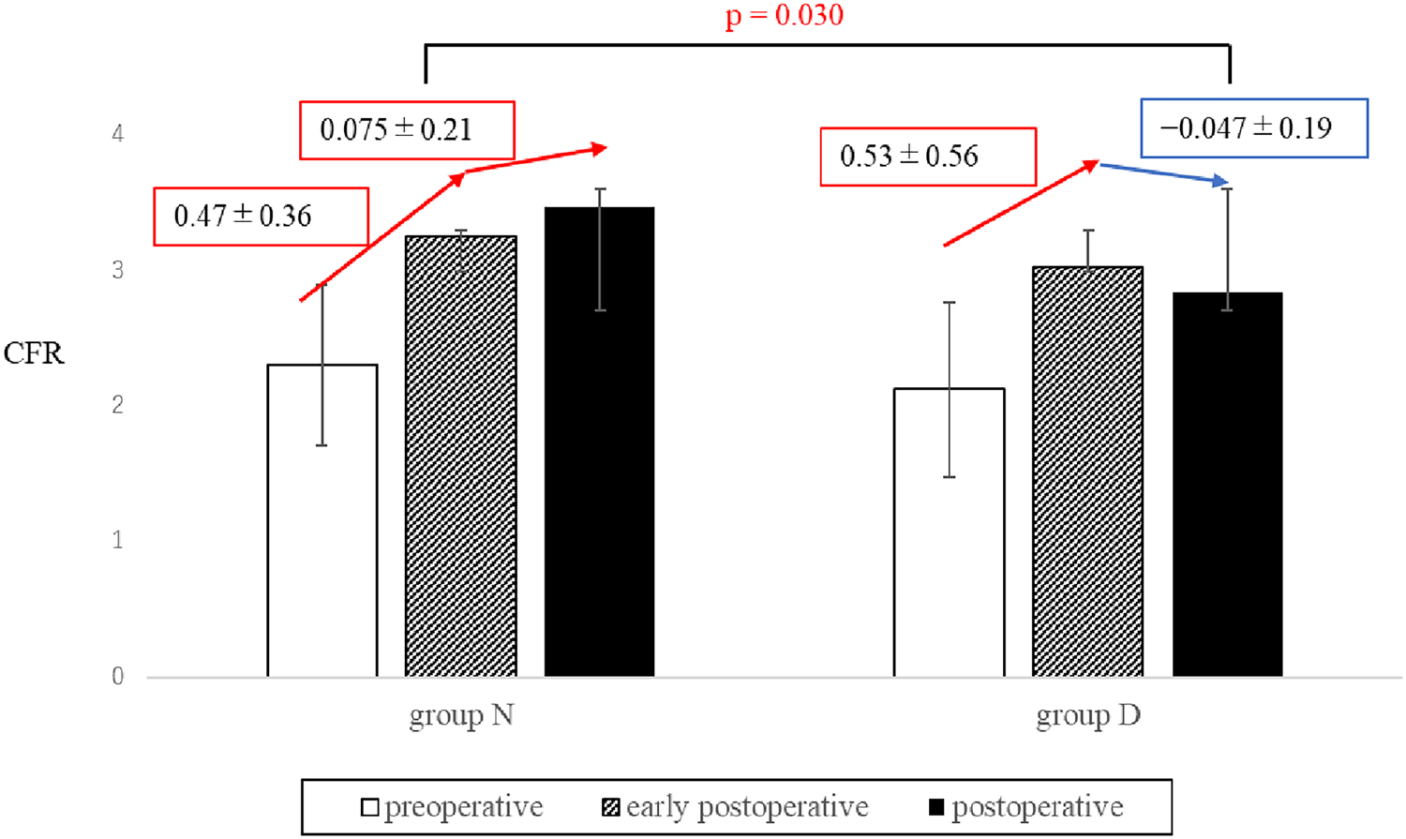
CFR data in the preoperative, early postoperative, and late postoperative periods in each group (arrows indicate CFR improvement). CFR: coronary flow reserve

**Fig. 2:**
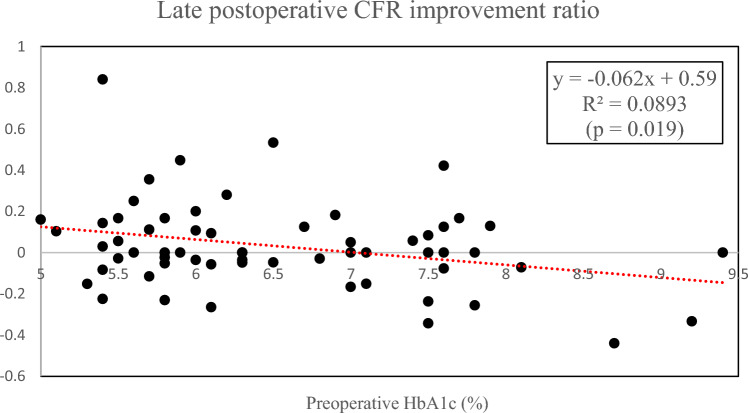
Relationship between preoperative HbA1c and late postoperative CFR improvement ratio. CFR: coronary flow reserve, HbA1c: hemoglobin A1c

Table 4 shows the CFR in the preoperative, early postoperative, and late postoperative periods. Preoperative CFR did not differ between the two groups (*p* = 0.29). CFR improved in both groups after CABG surgery, but there was no significant difference between the two groups in the early postoperative CFR (*p* = 0.63) or in the early postoperative CFR improvement ratio (*p* = 0.64). The mean time point of CFR evaluation in the late postoperative period was 26.1 ± 23.7 months in Group N and 23.6 ± 19.3 months in Group D (*p* = 0.69). In the late postoperative period, CFR in Group N was significantly higher than in Group D (p < 0.05). The late postoperative CFR improvement ratio in Group D was significantly lower than in Group N (*p* < 0.05) (Fig. 1). Furthermore, late postoperative CFR was significantly correlated with the preoperative HbA1c concentration (*R*^*2*^ = 0.13, *p* = 0.019) (Fig. 2).

**Table 4:** Preoperative, early postoperative, and late postoperative CFR data

	Group N	Group D	*p* value
CFR			
Preoperative	2.3 ± 0.59	2.1 ± 0.67	0.29
Early postoperative	3.3 ± 0.64	3.0 ± 0.72	0.63
Late postoperative	3.5 ± 0.80	2.9 ± 0.71	0.012
CFR improvement ratio			
Early postoperative CFR improvement ratio	0.47 ± 0.36	0.53 ± 0.56	0.64
Late postoperative CFR improvement ratio	0.075 ± 0.21	− 0.047 ± 0.20	0.030

Data are presented as mean ± standard deviation

*CFR* coronary flow reserve

Information on CFR measurements has been compiled in Supplementary Tables S1–3.

### Classification by history of DM

**Fig. 3:**
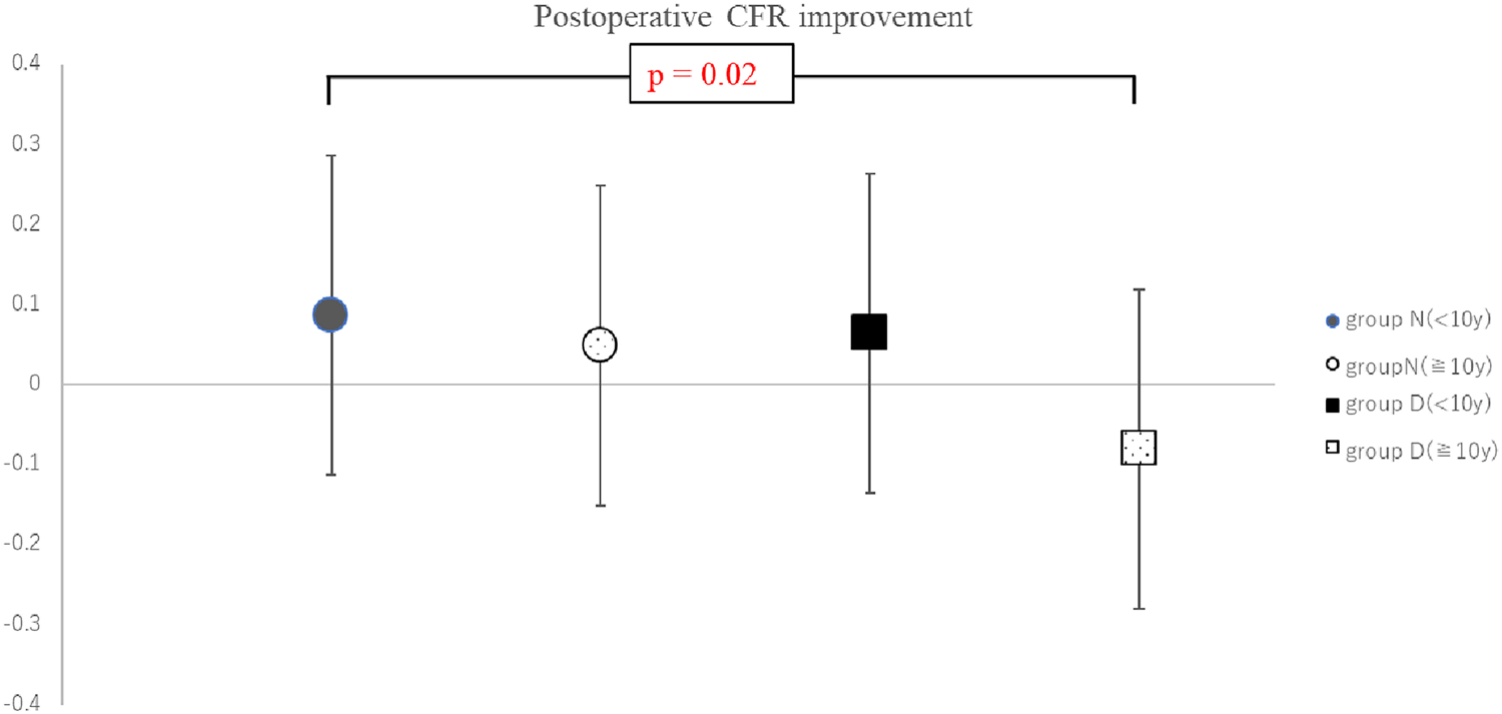
Multiple-comparison data of postoperative CFR improvement in both groups classified by DM history (< 10 years or ≥ 10 years). CFR: coronary flow reserve, DM: diabetes mellitus

Each group was also classified by the history of DM (≥ 10 years or < 10 years) (Table 5). Comparison of the four groups revealed that the patients in Group D with a DM history of ≥ 10 years had a significantly less pronounced improvement in late CFR than those in Group N with a DM history of < 10 years (*p* = 0.02) (Fig. 3).

**Table 5:** Late postoperative CFR improvement ratio in both groups classified by the history of DM

History of diabetes mellitus	Group N		Group D	
	< 10 y	≥ 10 y	< 10 y	≥ 10 y
n	29	9	5	17
Late postoperative CFR improvement ratio	0.087 ± 0.22	0.049 ± 0.20	0.064 ± 0.20	− 0.080 ± 0.18

Data are presented as mean ± standard deviation

*CFR* coronary flow reserve, *DM* diabetes mellitus

### Multivariate analysis

The multiple regression analysis revealed that preoperative HbA1c significantly affected late postoperative CFR improvement (*p* = 0.038) (Table 6). Additionally, logistic regression analysis was performed to identify factors associated with a lack of CFR improvement in the late postoperative period; however, no significant predictors were found (Table 7).

**Table 6:** Multiple regression analysis

Variable	*β*	SE	*t*-value	*p*-value
LDL-C	0.00047	0.00083	0.57	0.57
LV mass index	− 0.0014	0.0016	− 0.89	0.38
Ejection fraction	− 0.00047	0.0042	− 0.11	0.91
Preoperative HbA1c	− 0.060	0.028	− 2.13	0.038

*β* regression coefficient, *SE* standard error, *LDL-C* low-density lipoprotein cholesterol, *LV* left ventricular, *HbA1c* hemoglobin A1c

**Table 7:** Univariable logistic regression analysis

	Univariable logistic regression analysis		Observed event rate
	OR (95% CI)	*P* value	Positive/negative, *n* (%)
Preoperative data			
Male sex	3.18 (0.92, 11.0)	0.069	47 (77)/14 (23)
Hypertension	1.38 (0.18, 10.5)	0.76	57 (93)/4 (7)
DLP	2.27 (0.54, 9.6)	0.26	11 (18)/50 (43)
CKD	1.36 (0.47, 4.0)	0.57	21 (34)/40 (66)
Diabetes	2.04 (0.68, 6.1)	0.2	22 (36)/39 (64)
Ejection fraction of < 50%	1.74 (0.55, 5.5)	0.35	18 (30)/43 (70)
LVH	1.14 (0.29, 4.5)	0.85	10 (16)/51 (84)
OMI	1.55 (0.26, 9.2)	0.63	6 (10)/55 (90)
Postoperative data			
Β-blocker	1.60 (0.56, 4.6)	0.38	39 (64)/22 (36)
ACEI/ARB	0.35 (0.11, 1.0)	0.059	20 (33)/41 (67)
Metformin	1.37 (0.30, 6.3)	0.69	8 (13)/53 (87)
DPP-4 inhibitor	1.67 (0.52, 5.3)	0.39	17 (28)/54 (72)
Insulin	1.58 (0.46, 5.4)	0.47	14 (23)/47 (77)
HbA1c improvement	0.94 (0.32, 2.8)	0.92	20 (33)/41 (67)

*OR* odds ratio, *CI* confidence interval, *DLP* dyslipidemia, *CKD* chronic kidney disease, *LVH* left ventricular hypertrophy, *OMI* old myocardial infarction, *ACEI/ARB* angiotensin-converting enzyme inhibitor/angiotensin receptor blocker, *DPP-4* dipeptidyl peptidase-4, *HbA1c* hemoglobin A1c. Definitions: *Hypertension*: requirement for or currently receiving antihypertensive treatment. *DLP*: low-density lipoprotein cholesterol concentration of ≥ 120 mg/dL. *CKD*: estimated glomerular filtration rate of < 60 mL/min/1.73 m^2^. *Diabetes mellitus*: HbA1c concentration of ≥ 7%. *LVH*: left ventricular mass index of ≥ 115 g/m^2^ (male), ≥ 95 g/m^2^ (female). *OMI*: OMI in the left anterior descending artery area diagnosed by echocardiography. *HbA1c improvement*: (late postoperative HbA1c − preoperative HbA1c) < 0

## Discussion

Patients with DM are known to develop microvascular complications, including neuropathy, nephropathy, and retinopathy, and a high HbA1c concentration reportedly increases the risk of these complications [15, 16]. DM is also known to cause microvascular impairment in the coronary arteries, and decreased CFR has also been reported [17, 18]. It is known that CFR improves after CABG, but there are no reports on whether differences in the preoperative HbA1c concentration make a difference in the remote postoperative period.

Why might preoperative HbA1c affect CFR improvement after CABG in the long term? It is reasonable to speculate that factors such as postoperative glycemic control, medications affecting microcirculation, and lifestyle or exercise habits could influence outcomes in the late postoperative period. However, confirming this would require a prospective study. In this retrospective study, we examined the effects of diabetes control and related medications, but no significant contributing factors were identified. It is possible that in patients with poorly controlled or advanced DM, the degree of microvascular damage is such that revascularization does not sufficiently improve coronary microcirculation.

Our results first demonstrated that a preoperative HbA1c concentration of > 7% was an indicator of CFR improvement in the late postoperative period. The patients with a preoperative HbA1c concentration of > 7% may have already been exposed to coronary microcirculation impairment. It is known that hyperglycemia induces vascular endothelial dysfunction in patients with DM. Endothelial dysfunction is one of the causes of coronary microcirculatory impairment. As a result, these patients may have poor improvement in the coronary microcirculation in the remote postoperative period. In light of these results, an important question arises: what should we do for patients with poor preoperative glycemic control? Further studies are needed to investigate this issue, but medication therapy to improve vascular endothelial function may be recommended. For example, sodium–glucose co-transporter-2 inhibitors have been reported to protect vascular endothelial function independently of glycemic control and to improve CFR in previous studies [19, 20]. Although these agents were not commonly used in our cohort and their effects could not be evaluated in this study, future research may clarify whether such medications can optimize outcomes after CABG in patients with elevated preoperative HbA1c levels. However, there are other causes of CFR decline. Because CFR is calculated as the coronary blood flow during coronary artery dilation divided by the resting blood flow, it is reduced in patients with cardiac hypertrophy (e.g., patients with aortic stenosis or hypertension) in whom the resting blood flow is increased [11]. In the present study, there was no significant difference in the left ventricular mass index to compare cardiac hypertrophy between the two groups.

### Limitations

This study has some limitations that should be considered. First, the patients with an HbA1c concentration of < 7% included patients without DM. In the future, it may be necessary to subdivide the data. That said, the impact of preoperative hyperglycemia on the remote postoperative period can be studied using the methods described in this study.

Second, although this study evaluated the impact on CFR in the late postoperative period, it did not include an assessment of postoperative diabetes control. Ideally, postoperative glycemic control should also be evaluated; however, this is difficult in a retrospective study such as ours. Glycemic control can vary depending on both the attending physician and the patient, and in practice, HbA1c levels often fluctuate even within a single year. To accurately assess postoperative glycemic control, a prospective study would be required—an approach that was beyond the scope of the present study.

Finally, CFR is assessed by measuring the blood flow in the left anterior descending artery, but this does not necessarily reflect the microcirculation throughout the entire coronary system. Tests such as positron emission tomography scans can provide a more comprehensive evaluation of the coronary microcirculation; however, such scans are rarely performed at our hospital. Therefore, we adopted non-invasive measurement using echocardiography. Notably, CFR measurement by echocardiography is reportedly comparable to positron emission tomography in terms of accuracy [21].

## Conclusion

In the present study, the preoperative HbA1c concentration was associated with late postoperative CFR improvement after CABG. Patients with a high preoperative HbA1c concentration may require more adequate preoperative and postoperative glycemic control and pharmacologic therapy.

## Supplementary Information

Below is the link to the electronic supplementary material.

Supplementary file1 (DOCX 22 KB)

## Data Availability

The datasets generated and analyzed during the current study are available from the corresponding author on reasonable request.
