# Supplementary material for: Relationship between preoperative hemoglobin A1c and late postoperative coronary flow reserve improvement after coronary artery bypass grafting
Source: Gen Thorac Cardiovasc Surg. 2025 Aug 12;74(2):133–40. doi: 10.1007/s11748-025-02189-0 (PMC12913312; doi:10.1007/s11748-025-02189-0)
Supplement: Supplementary file 1 — Supplementary file1 (DOCX 22 KB) [file 11748_2025_2189_MOESM1_ESM.docx]

**Supplementary Tables**

**Relationship between preoperative hemoglobin A1c and late postoperative coronary flow reserve improvement after coronary artery bypass grafting**

*General Thoracic and Cardiovascular Surgery*

Takahiro Fujimoto, Kentaro Honda, Hideki Kunimoto, Ryo Nakamura, Mizuho Ikuchi, Yuya Ideguchi, Kota Agematsu, Yoshiharu Nishimura

Corresponding author

Takahiro Fujimoto, MD

Department of Thoracic and Cardiovascular Surgery, Wakayama Medical University, 811-1

Tel: +81(0)-73-424-5185, FAX: +81(0)-73-446-4761

Email: taka46.fuji@gmail.com

**Table S1.** Preoperative CFR data

| Preoperative CFR data | | Group N (n = 39) | Group D (n = 22) | p value |
| --- | --- | --- | --- | --- |
| CFR | | 2.3 ± 0.60 | 2.1 ± 0.70 | 0.29 |
| LAD flow, m/s | Rest | 21.9 ± 7.1 | 20.6 ± 8.2 | 0.53 |
|  | Hyperemia | 51.0 ± 16.9 | 42.5 ± 16.6 | 0.064 |
| Systolic BP, mmHg | Rest | 131 ± 17.0 | 132 ± 19.0 | 0.84 |
|  | Hyperemia | 122 ± 21.8 | 121 ± 18.77 | 0.95 |
| Diastolic BP, mmHg | Rest | 75.4 ± 11.8 | 73.5 ± 12.7 | 0.56 |
|  | Hyperemia | 67.6 ± 10.9 | 68.6 ± 12.0 | 0.33 |
| HR, beats/min | Rest | 60.7 ± 9.7 | 61.9 ± 9.4 | 0.73 |
|  | Hyperemia | 67.6 ± 10.9 | 67.5 ± 10.4 | 0.98 |

Data are presented as mean ± standard deviation.

CFR: coronary flow reserve, LAD: left anterior descending artery, BP: blood pressure, HR: heart rate

**Table S2.** Early postoperative CFR data

| Early postoperative CFR data | | Group N (n = 39) | Group D (n = 22) | p value |
| --- | --- | --- | --- | --- |
| Duration from CABG to CFR measurement, months | | 2.6 ± 3.4 | 2.1 ± 2.7 | 0.54 |
| CFR | | 3.3 ± 0.65 | 3.2 ± 0.61 | 0.63 |
| Postoperative CFR improvement | | 0.47 ± 0.36 | 0.53 ± 0.57 | 0.64 |
| LAD flow, m/s | Rest | 18.7 ± 6.2 | 19.9 ± 4.5 | 0.44 |
|  | Hyperemia | 61.4 ± 24.3 | 62.6 ± 16.3 | 0.85 |
| Systolic BP, mmHg | Rest | 121.8 ± 27.1 | 123.1 ± 16.9 | 0.84 |
|  | Hyperemia | 116.8 ± 21.0 | 115.6 ± 15.4 | 0.81 |
| Diastolic BP, mmHg | Rest | 75.6 ± 12.4 | 69.5 ± 8.9 | 0.057 |
|  | Hyperemia | 68.0 ± 12.0 | 65.0 ± 9.2 | 0.33 |
| HR, beats/min | Rest | 63.0 ± 10.4 | 65.4 ± 10.0 | 0.40 |
|  | Hyperemia | 72.5 ± 10.2 | 71.0 ± 10.3 | 0.58 |

Data are presented as mean ± standard deviation.

CFR: coronary flow reserve, CABG: coronary artery bypass grafting, LAD: left anterior descending artery, BP: blood pressure, HR: heart rate

**Table S3.** Late postoperative CFR data

| Late postoperative CFR data | | Group N (n = 39) | Group D (n = 22) | p value |
| --- | --- | --- | --- | --- |
| Duration from CABG to CFR measurement, months | | 26.1 ± 23.7 | 23.6 ± 19.3 | 0.69 |
| CFR | | 3.5 ± 0.80 | 2.9 ± 0.68 | 0.012 |
| Late postoperative CFR improvement | | 0.075 ± 0.21 | −0.047 ± 0.20 | 0.030 |
| LAD flow, m/s | Rest | 20.3 ± 4.9 | 20.0 ± 6.6 | 0.85 |
|  | Hyperemia | 70.5 ± 24.2 | 57.3 ± 16.1 | 0.033 |
| Systolic BP, mmHg | Rest | 124.7 ± 26.0 | 128.7 ± 18.1 | 0.54 |
|  | Hyperemia | 119.3 ± 18.5 | 120.0 ± 18.1 | 0.90 |
| Diastolic BP, mmHg | Rest | 73.7 ± 9.5 | 74.3 ± 9.2 | 0.82 |
|  | Hyperemia | 68.7 ± 10.2 | 70.0 ± 10.5 | 0.77 |
| HR, beats/min | Rest | 61.8 ± 8.6 | 68.0 ± 9.8 | 0.016 |
|  | Hyperemia | 70.7 ± 10.2 | 73.0 ± 10.5 | 0.42 |

Data are presented as mean ± standard deviation.

CFR: coronary flow reserve, CABG: coronary artery bypass grafting, LAD: left anterior descending artery, BP: blood pressure, HR: heart rate

**Additional data**

| Regression statistics | |
| --- | --- |
| Multiple correlation coefficient (R) | 0.3283379 |
| Coefficient of determination (R^2^) | 0.1078058 |
| Adjusted R^2^ | 0.0440776 |
| Standard error | 0.2083719 |
| N | 61 |
